# Multiome in the Same Cell Reveals the Impact of Osmotic Stress on *Arabidopsis* Root Tip Development at Single‐Cell Level

**DOI:** 10.1002/advs.202308384

**Published:** 2024-04-18

**Authors:** Qing Liu, Wei Ma, Ruiying Chen, Shang‐Tong Li, Qifan Wang, Cai Wei, Yiguo Hong, Hai‐Xi Sun, Qi Cheng, Jianjun Zhao, Jingmin Kang

**Affiliations:** ^1^ State Key Laboratory of North China Crop Improvement and Regulation Key Laboratory of Vegetable Germplasm Innovation and Utilization of Hebei Ministry of Education of China‐Hebei Province Joint Innovation Center for Efficient Green Vegetable Industry International Joint R & D Center of Hebei Province in Modern Agricultural Biotechnology College of Life Sciences College of Horticulture Hebei Agricultural University Baoding 071000 China; ^2^ BGI Research Beijing 102601 China; ^3^ BGI Research Shenzhen 518083 China; ^4^ College of Life Sciences University of Chinese Academy of Sciences Beijing 100049 China; ^5^ Glbizzia Biosciences Beijing 102609 China; ^6^ School of Life Sciences University of Warwick Coventry CV4 7AL UK

**Keywords:** *Arabidopsis* root tip, osmotic stress, single‐nucleus multi‐omics, transcriptional regulatory networks

## Abstract

Cell‐specific transcriptional regulatory networks (TRNs) play vital roles in plant development and response to environmental stresses. However, traditional single‐cell mono‐omics techniques are unable to directly capture the relationships and dynamics between different layers of molecular information within the same cells. While advanced algorithm facilitates merging scRNA‐seq and scATAC‐seq datasets, accurate data integration remains a challenge, particularly when investigating cell‐type‐specific TRNs. By examining gene expression and chromatin accessibility simultaneously in 16,670 *Arabidopsis* root tip nuclei, the TRNs are reconstructed that govern root tip development under osmotic stress. In contrast to commonly used computational integration at cell‐type level, 12,968 peak‐to‐gene linkage is captured at the bona fide single‐cell level and construct TRNs at an unprecedented resolution. Furthermore, the unprecedented datasets allow to more accurately reconstruct the coordinated changes of gene expression and chromatin states during cellular state transition. During root tip development, chromatin accessibility of initial cells precedes gene expression, suggesting that changes in chromatin accessibility may prime cells for subsequent differentiation steps. Pseudo‐time trajectory analysis reveal that osmotic stress can shift the functional differentiation of trichoblast. Candidate stress‐related gene‐linked cis‐regulatory elements (gl‐cCREs) as well as potential target genes are also identified, and uncovered large cellular heterogeneity under osmotic stress.

## Graphical abstract

Plant responses to environmental stress are controlled by transcriptional regulatory networks (TRNs). Here, the cell type‐specific TRNs are reconstructed in *Arabidopsis* root tip by examining gene expression and chromatin accessibility simultaneously in 16,670 nuclei, and elucidated the power of single‐cell multiome approach in uncovering the mechanisms of osmotic stress response.

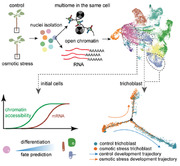

## 1 Introduction

Sessile plants have evolved various mechanisms to adapt, survive, and thrive under constantly changing climates. For instance, specific cell and tissue differentiation may facilitate plants to cope with stress environments, and such responses often depend on selective gene expression during plant growth and development.^[^ ^1^, ^2^ ^]^ Gene expression occurs only when the gene‐associated chromatin becomes accessible by transcription machinery.^[^ ^3^ ^]^ The process of cell differentiation, coupled with subsequent tissue and organ development and environmental changes can alter the dynamics of chromatin accessibility, ultimately leading to gene expression specific to each cell type.^[^ ^3^, ^4^, ^5^ ^]^ Hence, having the global RNA transcript profile and genome‐wide chromatin accessibility in single cells is essential to unravel transcriptional regulatory networks (TRNs) that modulate how cell differentiates, how tissue and organ develop and how plants respond to biotic and abiotic environmental stresses.

Single‐cell RNA sequencing (scRNA‐seq) enables the detection of gene expression in individual cells,^[^ ^6^, ^7^, ^8^, ^9^, ^10^ ^]^ while single‐cell ATAC sequencing (scATAC‐seq) provides access to open chromatin data for each cell.^[^ ^11^, ^12^ ^]^ The concurrent application of both technologies can establish a relationship between gene expression and regulatory element accessibility at the single‐cell level, promoting the discovery of underlying transcriptional regulatory mechanisms and more accurately reconstructing fundamental physiological cellular processes.^[^ ^13^, ^14^, ^15^ ^]^ Most simultaneous single‐cell gene expression and chromatin accessibility studies involve conducting separate scRNA‐seq and scATAC‐seq experiments on the same samples, then integrating the two sets of omics data using algorithms to link them and “anchor” homogenous cells across the data sets.^[^ ^16^, ^17^, ^18^, ^19^ ^]^ However, while data integration methods can achieve single‐cell gene expression and chromatin accessibility analysis, they do not allow identifying specific relationships between the two data types for each cell, because accurate integration of multi‐omics data remains to be challenging.^[^ ^20^ ^]^ Consequently, chromatin accessibility and RNA expression within the same cell are necessary to directly correlate transcriptional regulation data with its RNA products, allowing for a more accurate and comprehensive description of single‐cell types and developmental statuses.^[^ ^20^, ^21^ ^]^ Although this technology has been widely applied in human and animal research, it remains largely unexplored in the field of plants.

Drought, high salt and low temperature lead to osmotic stress, resulting in the reduction of crop production and having an important impact on food security. However, the TRNs underlying osmotic stress responses in different cell types are still unknown.^[^ ^22^, ^23^ ^]^ In this study, we used the 10x Chromium Single Cell Multiome ATAC + Gene Expression platform to simultaneously perform transcriptome and chromatin accessibility sequencing within the same cell in *Arabidopsis* root tips. This approach enabled us to more precisely dissect the TRNs of *Arabidopsis* gene expression under osmotic stress at the single‐cell level.

## 2 Results

### 2.1 Multiple Omics in the Same Cell Generates High‐Quality Genome‐Wide Chromatin and Expression Profiles Across Cell Types

**Figure 1:**
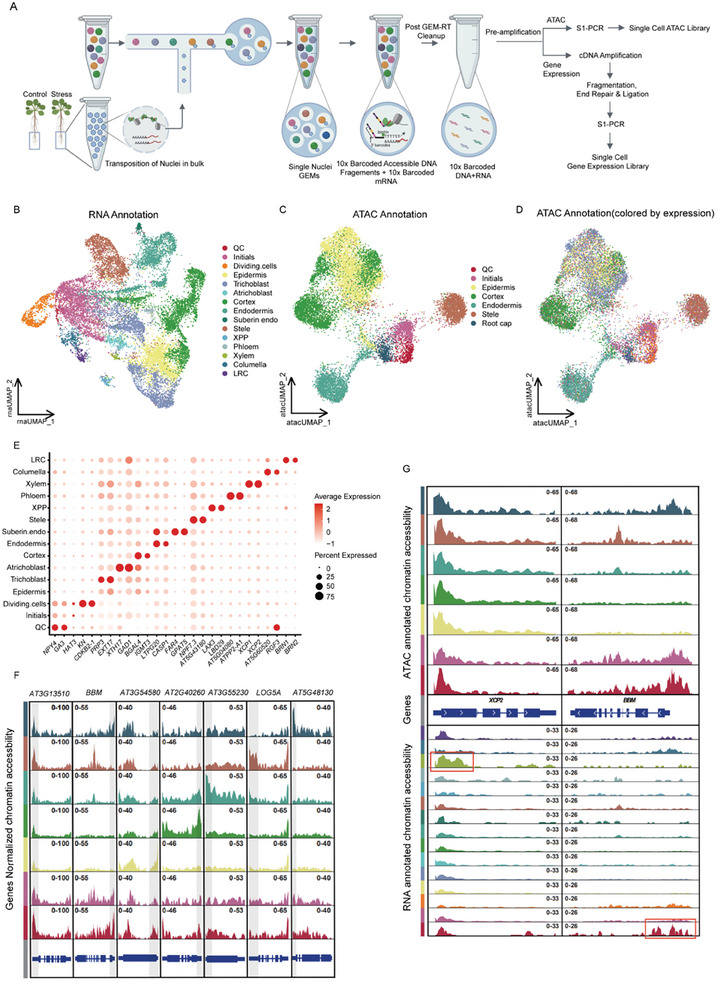
Multiple Omics in the same cell generates high‐quality genome‐wide chromatin and expression profiles across cell types. A) Overview of *Arabidopsis* root tips single‐cell multi‐omics workflow. B) UMAP visualization of 16,670 single cells derived from *Arabidopsis thaliana* root tips annotated by RNA expression. Cells colored by clusters were defined by RNA clustering, and cell types were defined using known markers, QC (quiescent center), XPP (xylem pole pericycle), LRC (lateral root cap). C) UMAP visualization of 16,670 single cells derived from *Arabidopsis thaliana* root tips defined by ATAC accessibility. Cells are labeled with results of independent annotation with chromatin accessibility data. D) The same UMAP projection as (C) with cells labeled by RNA annotation. E) Expression of known marker genes in each cell type. Dot size indicates the percentage of cells expressing the gene (% expressed). Dot colors represent the proportional amount of expression of each gene in each cell type, with warmer colors indicating higher expression levels. F) Chromatin accessibility of marker genes for each cell type with ATAC annotation, gray shading for marker gene promoter region. G) Chromatin accessibility of known markers *XCP2* (xylem) and *BBM* (QC) in RNA and ATAC annotation.

To investigate transcriptional dynamics in *Arabidopsis* root tips under osmotic stress at the single‐cell level, we extracted nuclei from 5‐mm root tip samples (10 days old) grown under normal culture conditions (1/2MS) and osmotic stress (1/2MS+250 mM sorbitol). Adding sorbitol to the medium led to typical osmotic stress phenotypes, such as shorter root length, closed stomata, and leaves Ca^2+^ increased in *Arabidopsis*, and it was used as an osmotic stress inducer in previous studies.^[^ ^24^, ^25^, ^26^, ^27^, ^28^, ^29^, ^30^ ^]^ We employed the 10x Chromium Single Cell Multiome ATAC + Gene Expression platform to simultaneously determine transcriptome (snRNA‐seq) and chromatin accessibility (snATAC‐seq) within the same cell (**Figure** **1**A). In order to monitor the reproducibility of the experiments and the reliability of the data, two biological replicates were included for both control and stress samples. We collected 16,670 nuclei (control: 5,696; stress: 10,974), with an average of 2,433 genes, 4,782 RNA UMI counts, 3,358 peaks and 3,865 ATAC UMI counts per cell. The above quality control statistics among 4 samples were comparable (Figure S1A, Supporting Information). We had also noted that the TSS (Transcription Start Site) scores, both under control and stress conditions, exhibited values suitable for subsequent analysis (Figure S1B, Supporting Information). We performed uniform manifold approximation and projection (UMAP) independently on snRNA‐seq and snATAC‐seq data, and identified 15 distinct cell types (using snRNA‐seq data individually; Figure 1B) and 7 distinct cell types (using snATAC‐seq data individually; Figure 1C), cell types were assigned on the basis of known markers (Figure 1E,F; Figures S2 and S3A, and Table S1, Supporting Information), consistent with previous studies.^[^ ^7^, ^8^, ^18^ ^]^ We also observed strong concordance in transcriptome and chromatin accessibility between biological replicates (Figure S3B,C, Supporting Information). Subsequently, we assessed the correlation between transcriptomic and epigenomic data using cell‐specific IDs (10x barcodes) and found that snRNA‐seq and snATAC‐seq matched in most cell types, indicating the high reliability of the data obtained from the same cells (Figure 1D).

We found that using ATAC annotation alone was unable to identify rare cell types as previous studies (e.g., xylem and phloem; Figure 1C).^[^ ^18^ ^]^ For instance, *BBM* and *XCP2*, which were markers of QC and xylem,^[^ ^18^, ^31^ ^]^ exhibited cell type‐restricted expression. We could not observe the specificity of their accessibility peaks among different cell types using ATAC annotation; however, it could be observed using RNA annotation (red box, Figure 1G). By analyzing the correlation of gene activity from snATAC‐seq and gene expression from snRNA‐seq among 15 cell types, we found that the correlation of rare cell types was lower than other cell types, this could be due to ATAC‐seq signals from highly abundant cells overshadowing signals from rare cell types (Figure S2A–C, Supporting Information).^[^ ^21^ ^]^ This emphasizes the necessity of simultaneous profiling of RNA levels and chromatin accessibility within the same cell to obtain a more comprehensive and accurate characterization of the transcriptional and epigenetic landscape. Since the transcriptome could more directly reflect gene expression levels and was better suited for annotating cell types, in subsequent analyses, we utilized RNA annotation data to identify cell types, and obtained corresponding chromatin accessibility data by using cell‐specific IDs (10x barcodes).

### 2.2 Chromatin Accessibility and Gene Expression Level Differentially Marked Cellular State

**Figure 2:**
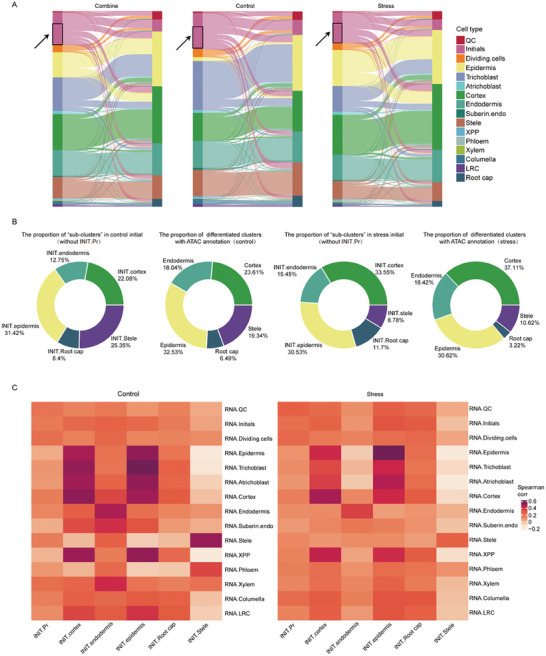
Chromatin accessibility and gene expression level differentially marked cellular state. A) Sankey diagrams of RNA‐ATAC correlation in combine, control and stress groups. RNA annotation was shown on the left and ATAC annotation was shown on the right of the Sankey diagrams. The initial cells marked with black boxes and arrows exhibited more open chromatin regions resembling differentiated cells. B) The proportion of differentiated “sub‐clusters” and clusters (ATAC annotation) in control and stress samples respectively. C) Spearman correlations between clusters (based on RNA expression) and “sub‐clusters” (based on gene accessibility) in control and stress samples respectively.

In order to visually observe the difference in chromatin accessibility between RNA annotation and ATAC annotation in each cell type, we plotted Sankey diagram of combined, control and stress groups respectively. The Sankey diagrams of the RNA‐seq and ATAC‐seq correlation analysis revealed an interesting phenomenon (**Figure** **2**A): most initial cells exhibited more open chromatin regions resembling differentiated cells (marked with black boxes and arrows in Figure 2A), however, the expression levels of these accessible genes in the initial cells remained low. By using chromatin accessibility data, we divided initial cells into six “sub‐clusters”: INIT.epidermis/cortex/endodermis/stele/root cap (initial cells exhibited more open chromatin regions resembling differentiated cells), and INIT.Pr (initial cells exhibited more open chromatin regions resembling primary cells such as QC and initial). Approximately 60.95% (control)/64.13% (stress) initial cells exhibited chromatin accessibility similar to differentiated cells (Figure S4A,B, Supporting Information). By analyzing the cell proportion, we found that the cell proportion of INIT.epidermis/cortex/endodermis/stele/Root cap was similar to the proportion of corresponding cell types under chromatin state classification, whether in the control or the stress samples (Figure 2B).

In addition, we calculated the gene scores of the six “sub‐clusters” based on ATAC‐seq peaks, and performed correlation analysis with 15 cell types (Figure 2C). In both control and stress samples, INIT.cortex/stele/endodermis exhibited the highest correlation with the corresponding cell type to be differentiated in the future, and INIT.Pr had a high correlation with QC, initials and other primary cells. In control samples, INIT.root cap had a higher correlation with columella than LRC, in contrast to the results of stress. INIT. epidermis had a high correlation with all the outermost cells, interestingly, INIT.epidermis(control) had the highest correlation with trichoblast, while INIT.epidermis (stress) had the highest correlation with epidermis, indicating that most of INIT.epidermis(control) might develop into trichoblast in the future, while most INIT.epidermis(stress) might develop into epidermis. In addition, microscopic observation showed that the number of trichoblast was reduced after 10 days of osmotic culture, compared with the control group (Figure S6A,B, Supporting Information), suggesting that the inhibition of trichoblast development by osmotic stress could be traced back to alterations in the initial cells (exhibited more open chromatin regions resembling epidermal cells), rather than only affecting the differentiation process from epidermis to trichoblast. Taken together, these results suggested that heterogeneity of chromatin accessibility among the initial cells were biologically significant and not due to errors in the analysis process.

### 2.3 Chromatin Accessibility Foreshadowed Future Cell Fate in Primary Cells

**Figure 3:**
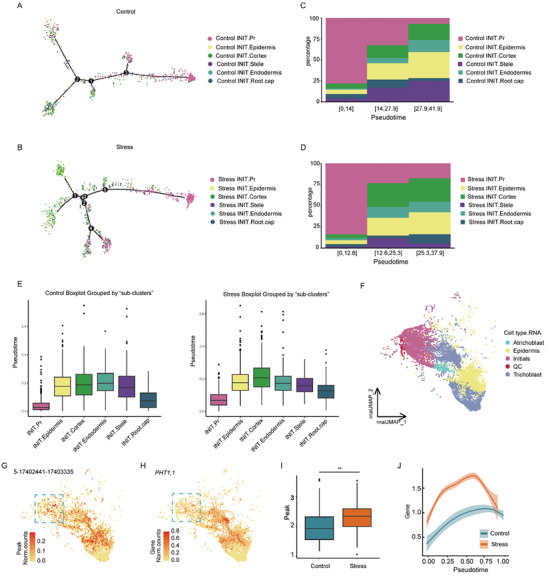
Chromatin accessibility foreshadowed future cell fate in primary cells. A,B) Developmental trajectories of control (A) and stress (B) initial cells (RNA annotation). Cells were colored based on gene accessibility. C,D) Proportional distribution of each “sub‐clusters” in the early, middle and late pseudotime. E) Box plot of pseudo‐time levels of six “sub‐clusters” under different conditions respectively. F) Pseudo‐time trajectory analysis of QC stem cells, initial, epidermis, atrichoblasts, and trichoblasts. G,H) Scaled chromatin accessibility (G) and RNA expression (H) patterns of *PHT1*. Initial, QC cells were marked with blue dashed lines. I) Box plot of peak accessibility under different conditions in initial epidermis lineage cells. J) *PHT1* expression levels in the stress and control groups over the pseudo‐time.

We constructed initial cell developmental trajectories using monocle2 and plotted the cells based on chromatin accessibility typing. Developmental trajectories showed that INIT.Pr was mainly concentrated at the beginning of trajectory, and those cells with chromatin accessibility similar to differentiated cells were located at the tail end of the trajectory (**Figure** **3**A,B; Figure S4C,D, Supporting Information). We divided the developmental trajectory into three stages along pseudo‐time order and calculated the proportion of each “sub‐clusters” in each stage. Proportion of INIT.Pr was very high in the early stages of development, ≈75%, and gradually decreased in the middle and late stages, accompanied by the gradual increase of differentiated “sub‐clusters” (Figure 3C,D). However, the expression level of initial marker genes did not change significantly throughout the developmental trajectory, suggesting that these cells were similar at the transcriptome level but differed in chromatin accessibility for subsequent differentiation programs (Figure S4E,F, Supporting Information). To further confirm such speculation, the differentiation order of the six “sub‐clusters” was analyzed using CytoTRACE in control and stress samples, respectively. We found that in the control and stress groups INIT.Pr differentiation was significantly lower than the other “sub‐clusters” (Figure 3E). The results of pseudo‐time trajectory reconstruction by independent methods suggested that chromatin was turned on prior to the onset of gene expression during root tip development, possibly in preparation for the subsequent differentiation step, a phenomenon that could only be detected by measurements of chromatin state and gene expression profile in the same cell. The analysis of single‐nuclei multiome data provided novel insights in explaining the inconsistency between chromatin state and gene expression at cell type level.

Among these chromatin regions that opened in advance, we identified the *PHT1;1* gene, participating in phosphorus absorption,^[^ ^32^ ^]^ with open chromatin in the outermost epidermal cells of roots and some initial cells (displaying similar chromatin accessibility to the epidermis). Phosphorus is one of the essential macronutrient elements for plant growth and development. Increasing the level of phosphorus nutrition could improve plant adaptation to drought and water use efficiency.^[^ ^33^ ^]^ As expected, *PHT1;1* mRNA was expressed in the outermost epidermal cells, however, *PHT1;1* was not expressed in initial cells despite its high chromatin accessibility in these cells. We employ CytoTRACE to track developmental trajectories in QC stem cells, initial cells, epidermal cells, atrichoblast, and trichoblast. QC cells were located at the beginning of the trajectory, with trichoblast at the end (Figure 3F; Figure S4G,H, Supporting Information). After simultaneous profiling of *PHT1;1* chromatin accessibility data and expression levels along the developmental trajectory, we observed that in some initial cells, chromatin was open for transcription initiation, but this gene was not expressed (Blue dotted line notation; Figure 3G,H). We also discovered that the accessible peak number and expression level of *PHT1;1* were higher in osmotic stress‐exposed cells than those in control cells (Figure 3I,J).

Subsequently, we focused on osmotic stress and auxin related genes that had been well‐studied in *Arabidopsis* (Table S2, Supporting Information) and investigated their expression patterns and chromatin accessibility across different cell types (Figure S4I, Supporting Information). We found that the gene expression and chromatin accessibility of auxin‐related genes were coordinately attenuated in a cell‐type specific manner in the stress group (Figure S4I, Supporting Information). For example, auxin synthesis related gene *YUC5* was suppressed in phloem, genes involved in auxin transport such as *ABCB19*, *PIN1* and auxin signaling related gene *IAA30* were specially suppressed in QC by osmotic stress. Additionally, osmotic stress related genes were cell‐type specifically induced in stress group, and the accessible peak number was higher in stress group than control group, i.e. the gene expression of ABA signaling pathway key regulators ABI5 was induced in xylem, another ABA signal transduction related protein ABI2 was specially induced in Suberin endodermis and XPP (Figure S4I, Supporting Information), suggesting the spatial control of the functional divergency of members in the same gene family. Interestingly, the expression levels of these stress related genes were specifically induced in only one or two cell types despite their chromatin were more accessible in more cell types, suggesting the activation of these genes were controlled by complex transcriptional regulation depending on the context of cell type.

### 2.4 Cell‐Type Specific Changes under Osmotic Stress

**Figure 4:**
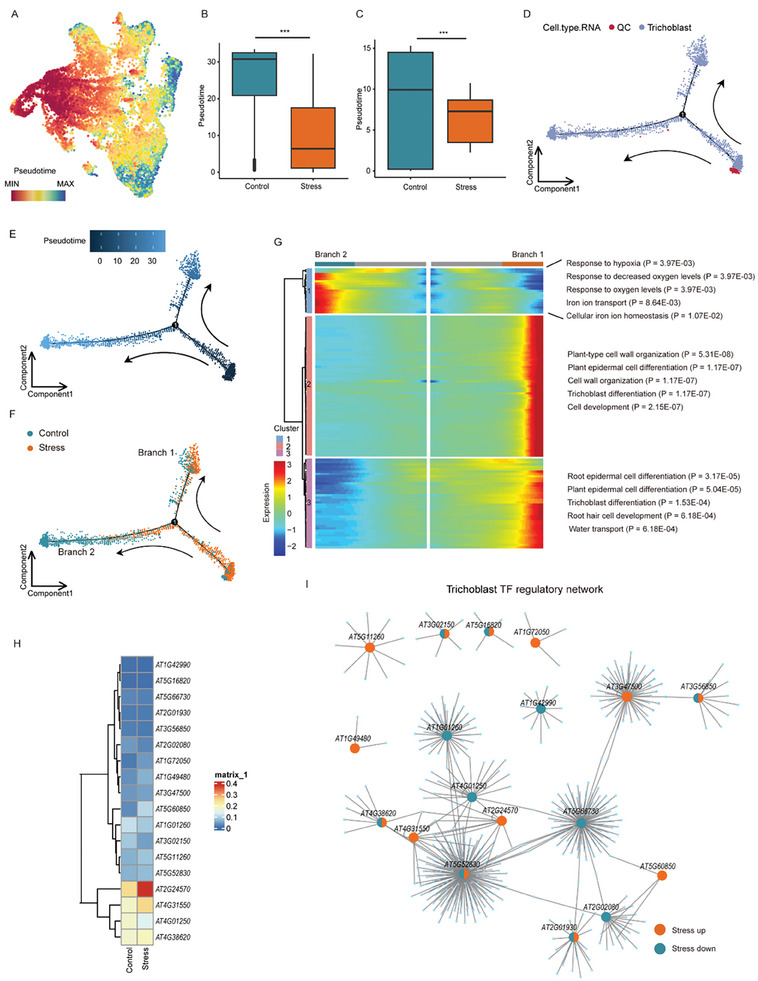
Cell‐type specific changes under osmotic stress. A) Root tip cells annotated with consensus pseudo‐time levels. B) Box plot showing pseudo‐time levels under different conditions in root tip cells. C) Box plot of pseudo‐time levels under different conditions in trichoblast. D–F) Developmental trajectories of QC and trichoblast. Cells were colored by control and stress conditions(F), pseudo‐time (E) and cell types (D). G) Heatmap showing the expression of regulatory genes on pseudo‐time developmental trajectories of trichoblast. The branch point in the middle indicates the beginning of the pseudo‐time. The three gene clusters determined by expression patterns were shown on the left, and the GO enrichment analyses of each gene cluster were shown on the right. The color bar indicates the relative expression level. H) Heatmap showing the entire regulon identified by SCENIC in trichoblast, with warmer colors indicating higher regulon activity. I) TFs regulatory networks showing the transcription factors regulation in trichoblast.

Osmotic stress triggers specific responses in gene expression, development, and plant physiology.^[^ ^1^ ^]^ We used CytoTRACE to track the developmental trajectories of *Arabidopsis* root cells from low to high differentiation. Combining this with RNA annotation results, we observed that trichoblast cells were enriched in the tail (indicating the end of differentiation), while QC cells were enriched in the start point (**Figure** **4**A; Figure S3D, Supporting Information). Control cells had higher pseudo‐time than stressed cells, suggesting a greater differentiation potential (Figure 4B). These results suggested that osmotic stress perturbed the physiological functions of various cell types in the *Arabidopsis* roots. Differential gene expression analysis showed that compared with the phloem, Suberin‐endo and XPP cells, there were more differentially expressed genes in trichoblast, cortex, endodermis and stele, under osmotic stress. These included genes that encoded osmotic stress related protein kinase, such as *MPK3* ^[^ ^34^ ^]^ and *SnRK2.8*,^[^ ^35^ ^]^ which were greatly induced in the cortex by osmotic stress (Figure S5B, Supporting Information). Encoding Na^+^ transporter gene *HKT1* ^[^ ^36^ ^]^ and the salt tolerance gene *AT1G13930* ^[^ ^37^ ^]^ were enriched in the stele under osmotic stress. We observed that another protein kinases SnRK2.7^[^ ^38^ ^]^ and the root halotropism‐associated protein SP2L^[^ ^39^ ^]^ were induced in trichoblast by osmotic stress (Figure S5B, Supporting Information). The above results suggested that these cell types might play a more crucial role in the response of root tip to osmotic stress. We performed GO enrichment analysis of the stress up‐regulated genes in each cluster, and selected the top5 terms for display (Figure S5C, Supporting Information). The expression levels of genes related to water transport and fluid transport were elevated in most cell types except trichoblast, cortex, endoderm, and stele. The cortex, endodermis, and stele cells showed similar responses to osmotic stress, and osmotic stress induced up‐regulation of genes associated with the generation of precursor metabolites and ATP metabolic process in these cells (Figure S5C, Supporting Information). Interestingly, trichoblast exhibited a specific response pattern to osmotic stress, with genes enriched in pathways related to aerobic respiration, cellular respiration and oxidative phosphorylation, suggesting that distinct mechanisms might act in response to external environmental factors in each cell type (Figure S5B, Supporting Information).

Trichoblast (root hair) was a crucial cell type in root tip for absorbing water, inorganic salts and sensing environmental changes,^[^ ^40^ ^]^ it had the largest number of differentially expressed genes compared with other cell types under osmotic stress (Figure S5A, Supporting Information). Through phenotypic observation, we also discovered that compared with the control, trichoblast in stress samples were reduced (Figure S6A,B, Supporting Information), therefore, we employed CytoTRACE to track developmental trajectories in trichoblast. CytoTRACE results showed that in the control group trichoblast differentiation was higher than the stress group (Figure 4C), consistent with the results in overall level (Figure 4B). Subsequently we analyzed the impact of osmotic stress on trichoblast development by reconstructing their pseudo‐time trajectories using both control and stressed cells. We used Monocle2 for pseudo‐time analysis of trichoblast and QC cells, and QC was defined as the initiation point of the starting position in the pseudo‐time trajectory (Figure 4D–F). Near the initiation point, the stress and control groups diverged along the same trajectory, forming two branches at bifurcation point 1: Branch 1 and Branch 2. We further found that based on expression changes across the trajectory, genes could be clustered into three clusters (Figure 4G). Genes in cluster 1 were highly expressed in Branch 2 and mainly regulated Branch 2 development, accompanied by the enrichment genes associated with hypoxia response‐related, iron transport and intracellular iron balance which constituted a large proportion of the control group (Figure 4G; Table S3, Supporting Information). In the developmental trajectory of Branch 1, which contained more trichoblast cells in the stress group, genes related to epidermal differentiation, trichoblast development, and water transport were highly expressed (cluster 2 and cluster 3) (Figure 4G; Table S3, Supporting Information).

Pseudotime trajectory reconstruction showed that there were two subtypes of trichoblast in *Arabidopsis* with distinct gene expression profiles: one of the subtypes consisted of mature trichoblast cells, which played an important role in responding to environmental changes and ion absorption (Branch2; Figure 4F); the other was the differentiating trichoblast cells (Branch1; Figure 4F), where most of the verified trichoblast marker genes were expressed (Figure S7A and Table S4, Supporting Information). The above results suggested that osmotic stress changed the physiological state of trichoblast, and inhibited the transition of partial trichoblast cells from differentiation to maturation, and thus affecting the physiological functions of trichoblast (Figure 4D–F).

Plants have evolved complex transcriptional regulatory networks (TRNs) to adapt to osmotic stress such as drought and high salinity, transcription factors (TFs) play a central role in TRNs by acting as molecular switches.^[^ ^41^ ^]^ Previous studies of TFs have primarily focused on whole tissue or organ levels, the trichoblast specific TF regulatory network remains largely unexplored.^[^ ^41^, ^42^, ^43^ ^]^ To address this gap, we investigated trichoblast trans‐regulatory elements under control and stress conditions, particularly focusing on the TFs responsive to osmotic stress. We employed SCENIC to analyze the expression of *Arabidopsis* TF families in trichoblast, and plotted their regulon activity heat maps and regulatory network (Figure 4H,I). Our analysis found that *WRKY17 (AT2G24570)*, a member of the plant‐specific WRKY family TF induced by abiotic stress (salt, mannitol, drought),^[^ ^43^ ^]^ was highly expressed in the trichoblast under osmotic stress (Figure 4H). Consistent with this, the motif accessibility of WRKY17 was increased in trichoblast (Figure S7B, Supporting Information). More importantly, we further observed that the target genes whose promoters contain WRKY17 binding motifs were upregulated in the stress group. In trichoblast regulatory network, we also identified *WRKY11(AT4G31550)*,^[^ ^43^ ^]^ which was phylogenetically related to *WRKY17* and was also induced by osmotic stress (Figure 4H). Notably, the motif accessibility and target gene expression pattern of WRKY11 exhibit similarities to WRKY17 (Figure S7C, Supporting Information). Moreover, *WRKY17* and *WRKY11* expression levels in control and osmotic‐treated root tips of *Arabidopsis* were detected by qPCR. We observed that the expression levels of *WRKY17* and *WRKY11* were higher in osmotic‐treated samples compared to the control (Figure S7D,E, Supporting Information). WRKY transcription factors play a crucial role in regulating plant responses to both biotic and abiotic stresses, the cell‐type specificity of WRKY transcription factors in response to abiotic stress remains incompletely understood, despite research on multiple plant species.^[^ ^44^, ^45^ ^]^ By employing single‐cell multi‐omics technology, we found that *WRKY11/17* was highly expressed in the trichoblast under stress and may play a critical role in the osmotic stress response.

### 2.5 Cell‐Type Specific *cis*‐Regulation of Transcription During Osmotic Stress

Gene expression in plants is regulated by *cis*‐regulatory elements (CREs), such as promoters and enhancers, which respond to developmental and environmental changes.^[^ ^46^ ^]^ Although substantial progress has been made in studying CREs in *Arabidopsis* at the tissue level,^[^ ^47^, ^48^ ^]^ a deeper understanding of cell‐type‐specific gene regulatory programs can be achieved using single‐cell multi‐omics technologies. By employing Single Cell Multiome ATAC + Gene Expression in the same cell, we aimed to identify cell‐specific gene‐linked candidate *cis*‐regulatory elements (gl‐cCREs) with improved accuracy. By analyzing snATAC‐seq data, we predicted *cis*‐regulatory interactions in the genome by detecting co‐accessible peak pairs, where a peak was located in the promoter region and chromatin accessibility was positively correlated with downstream gene expression.^[^ ^14^ ^]^ Accordingly, we predicted a set of CRE promoter pairs and identified 7,373 gl‐cCREs and 3,763 cCRE‐linked genes in pseudo‐bulk (Table S5, Supporting Information). Our analysis proved reliable, as demonstrated by the overlap between validated *Arabidopsis* root enhancer sequences^[^ ^49^ ^]^ and our gl‐cCREs, with our gl‐cCREs containing Super Enhancer (SE) sequences (Figure S8A, Supporting Information).

**Figure 5:**
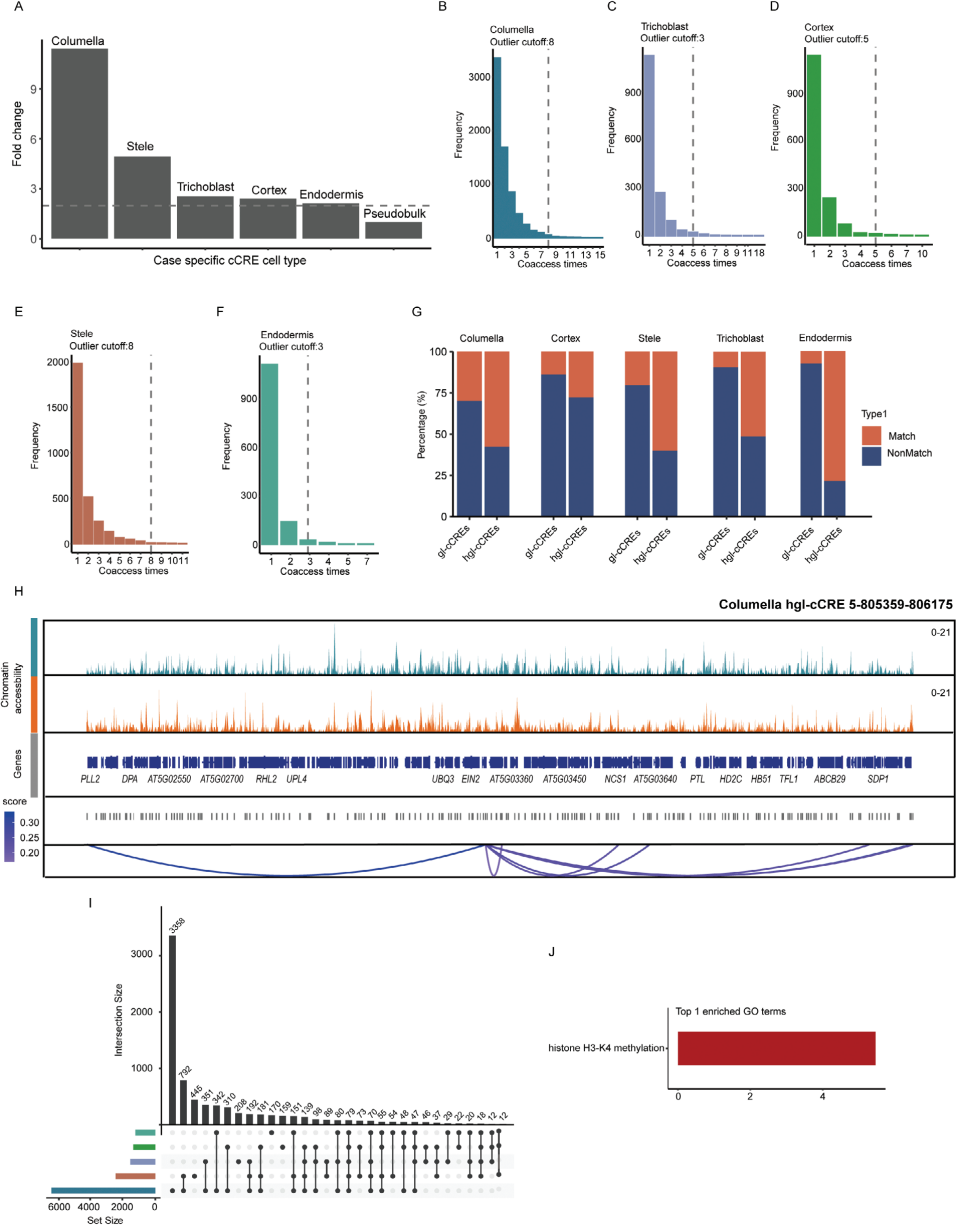
Cell‐type specific cis‐regulation of transcription during osmotic stress. A) Histogram showing fold changes of identified gl‐cCREs in different cell types and pseudo‐ bulk level. The gray dotted line represents the fold change of 2. B–F) Histogram showing frequency of gl‐cCREs coaccess times, the gray dotted lines indicate cutoff of hgl‐cCREs selection. G) Stacked histogram showing percentage of stress related gl‐cCREs and hgl‐cCREs matched with reported conserved noncoding sequences of crucifer regulatory regions. H) Accessibility level in different conditions and coaccessed peaks of columella hgl‐cCREs 5‐805359‐806175. I) Upset plot of target genes of stress related gl‐cCREs among different cell types. J) GO enrichment results of target genes unique in columella target genes. The x‐axis represent ‐log10 (*p*‐value).

Subsequently, we used snATAC‐seq data to predict stress‐related gl‐cCREs in different cell types (Table S5, Supporting Information) and found that, based on a fold‐change histogram, the number of stress‐related gl‐cCREs was much higher than that in pseudobulk, with the number of stress‐related gl‐cCREs in columella stress samples about ten‐fold higher than that in pseudo‐bulk (**Figure** **5**A). These results suggested that single‐cell multiome ATAC + Gene Expression allowed us to obtain more comprehensive information on stress‐cCREs, as some cell‐specific peaks were submerged and therefore unidentifiable under cell type level integration of snRNA‐seq and sATAC‐seq data.

We then focused on analyzing stress‐related gl‐cCREs with more interaction peak pairs than outliers, which were defined as stress‐related hot point gl‐cCREs (hgl‐CREs). In total, 240 hgl‐cCREs were found (Figure 5B–F; and Table S6, Supporting Information). Previous studies showed that enhancers have similar functions in different species and drive the cell‐specific expression of developmental genes.^[^ ^50^ ^]^ To explore the conservation of our hgl‐cCREs, we compared the gl‐cCREs and hgl‐cCREs sequences with published conserved noncoding sequences of the cruciferous family^[^ ^51^ ^]^ respectively. We found that most hgl‐cCREs contained multiple conserved noncoding sequences, and the proportion of hgl‐cCREs containing these sequences was significantly higher than that of gl‐cCREs (Figure 5G; and Table S7, Supporting Information). These results suggested that some conserved noncoding sequences might act as enhancer elements to regulate the expression of multiple genes in response to osmotic stress in *Arabidopsis* (Figure 5H; Figure S8H, Supporting Information), and these sequences might play similar roles in other cruciferous species, such as *Brassica rapa* and *Aethionema arabicum*.^[^ ^51^ ^]^

Given that the number of stress‐related gl‐cCREs and target genes in columella was significantly higher than other cell types (Figure 5A,I), we performed enrichment analysis on the target genes of stress‐related gl‐cCREs. Enrichment analysis indicated that these genes were associated with histone H3K4 methylation and were either core components of the histone H3K4 methylation complex^[^ ^52^ ^]^ or involved in the regulation of histone H3K4 methylation^[^ ^53^ ^]^ and other crucial biological processes (Figure 5J; and Table S8, Supporting Information). *Cis*‐regulatory element analysis showed that stress‐related gl‐cCREs were mainly involved in activating H3K4 methylation‐related gene expression in response to osmotic stress in columella. Simultaneously we examined the changes in histone methylation in response to osmotic stress, the bulk level of H3K4me3 was detected using CUT&Tag technology in the control and stress groups. Sequencing data demonstrated that the biological replicate samples were consistent and reliable, and the modification level significantly increased under osmotic stress (Figure S8C, Supporting Information). Enrichment analysis of H3K4me3‐modified genes revealed that H3K4me3‐related genes involved in oxidative phosphorylation, iron transport, jasmonic acid synthesis, and injury response were upregulated under osmotic stress (Figure S8D and Table S9, Supporting Information). A regulatory relationship may indeed exist between columella‐specific *cis*‐regulatory elements and H3K4 modification; nevertheless, the exact mechanism necessitates more detailed examination.

## 3 Discussion

There have been few studies on the effects of biotic and abiotic stresses on plants at the single‐cell level because protoplasts are most typically used in plant single‐cell research, however, the protoplasts preparation process is time consuming under harsh conditions, which cannot faithfully reflect the responses under stress. Compared with protoplasts, the preparation process for the nucleus was shorter, and the insufficient separation situation was avoided.^[^ ^17^ ^]^ In addition, it is necessary to prepare superior‐ quality nuclei samples to obtain transcriptome and chromatin accessibility information in the same cell, because transposase treatment is required before preparing the oil in well.

Here, we performed single nucleus ATAC‐seq and RNA‐seq simultaneously in the same cell, which was the first bona fide “single‐cell multiome” in plants (see Note added in proof). By simultaneously capturing chromatin accessibility and transcriptome data in the same cell, we discovered that chromatin accessibility predicted cell fate decisions in primary cells. This interesting phenomenon was not analyzed until present in plants, due to the difficulties in simultaneous capture of chromatin accessibility and mRNA level (Figure 3). RNA velocity utilized the differences between nascent and mature RNA (classified by intron and exon reads) to predict the cell fate,^[^ ^54^ ^]^ while chromatin accessibility changed before the nascent transcriptome was generated, and predicted cell fates prior to nascent transcription.

Single‐cell multi‐omics analysis enabled unprecedented high‐resolution view of how plant roots cope with osmotic stress. Our single‐cell analysis revealed that the response of *Arabidopsis* root tip to osmotic stress was both tissue‐specific and intricate, with trichoblast exhibiting a more distinct pattern of response compared to other cell types. The physiological state of *Arabidopsis thaliana* trichoblast was shifted under osmotic stress conditions. The population of mature trichoblast involved in ion absorption and environmental response decreased, while the number of initially differentiated trichoblast increased. Osmotic stress potentially induced the transition from initial differentiation to maturation in root hairs, thereby impacting plant development.

In this study, using Single‐Cell Multi‐omics ATAC + Gene Expression, the gene regulatory network across cell types in the root tip of *Arabidopsis* was analyzed under osmotic stress. Chromatin accessibility of the initials was similar to that of more differentiated cell types, indicating that stem cell chromatin would open up early during plant development to prepare for the next step of differentiation; such pattern was not well analyzed until present, due to the difficulties in simultaneous capture of chromatin accessibility and mRNA level. From the cell‐type specific single nucleus data, we found different cell types cope with osmotic stress differentially, either by specific TRN mediated by transcription factor, such as the trichoblast, or by stress‐related gl‐cCREs to adapt to osmotic stress, such as the columella. These findings helped to understand the complexity of dynamic molecular responses to osmotic stress, and contribute to the research of other abiotic stresses such as drought and salt stress.

Note added in proof: While this work was under revision, another research associated with multi‐omics sequencing of the same cell in plants was published elsewhere.^[^ ^55^ ^]^

## 4 Experimental Section

### Plant Material and Growth Conditions

*Arabidopsis thaliana* ecotype Col‐0 was grown in solid 1/2MS (control) and 1/2MS+250 mm sorbitol (stress) plates for 10 days (21 °C with 16 h light/8 h dark cycles, with light intensity of 300 lux).^[^ ^24^ ^]^

### Nucleus Isolation

The nuclei isolation procedure was modified from the product information sheet of the CelLyticTM PN Isolation/Extraction Kit (Sigma). Briefly, *Arabidopsis* root tips (5 mm) were chopped and mixed into 1× NIBTA buffer (1× NIB, 1 mm dithiothreitol, 1× ProtectRNA RNase inhibitor, 1× cOmplete, ethylenediaminetetraacetic acid‐free Protease Inhibitor Cocktail, and 0.3% Triton X‐100) on prechilled plates, and homogenates transferred to 15 mL tubes. Tubes were then shaken for 5 min on ice, after which lysates were passed through 40 µm strainers, and flow‐throughs were collected into new 15 mL tubes. These were then centrifuged for 10 min at 1,260 × *g*, supernatants decanted, and pellets resuspended in 4 mL of 1× NIBTA buffer. In new 15 mL tubes containing 80% Percoll solution (4 mL Percoll plus 1 mL NIBTA buffer), lysates were carefully overlaid onto Percoll layers. Tubes were centrifuged for 30 min at 800 × *g*, after which most of the nuclei had banded at the 1× NIBTA buffer and Percoll interface. Nuclei bands were gently collected into new 15 mL tubes, 10 mL of 1× NIBTA buffer was added, and the tubes were recentrifuged for 5 min at 1260 × *g*. Nuclei pellets were then washed twice in 1× NIBTA buffer, resuspended in diluted nuclei buffer (10× Genomics) to a final concentration of 3,000 nuclei uL^−1^, and then used as an input for single cell multiome ATAC plus gene expression library preparation (15,000 nuclei in 5 uL).

### Single Cell Multiome ATAC + Gene Expression and CUT&Tag Library Construction and Sequencing

The single cell multiome ATAC + gene expression libraries following the 10x Genomics Chromium Next GEM Single Cell ATAC Reagent Kits v2 protocol and sequenced on an Illumina NovaSeq 6000 paired‐end sequencing run (2 × 150 bp). The CUT&Tag library preparation was performed using the Vazyme Hyperactive Universal CUT&Tag Assay Kit. The amplification primers (Forward primer: TGTGAGCCAAGGAGTTGTTGTCTTCNNNNNNNNNNGTCTCGTGGGCTCGG, Reverse primer: GAACGACATGGCTACGATCCGACTTTCGTCGGCAGCGTC) were modified. Sequenced on an MGI 2000 paired‐end sequencing run (2 × 100 bp).

### Data Generation

Multiome matrices were generated using the 10x Cell Ranger ARC pipeline (2.0.2) with TAIR10 genome. The raw FASTQ files were first prepared from the sequencing library. The libraries were sequenced with Illumina NovaSeq 6000 under the modes of PE150 (scRNA‐seq library) and PE50 (scATAC‐seq library) by Glbizzia Biosciences. The non_nuclear_contigs parameter was then set to Mt and Pt in cellranger‐arc mkref process to avoid peak calling on contigs without chromatin structure. Finally, quantification was achieved using cellranger‐arc count.

### Data Processing

The “reduce” function was used from R package GenomicRanges^[^ ^56^ ^]^ (1.44.0) to merge all intersecting peaks from different batches. ATAC assay for each cell was created using R package Signac^[^ ^57^ ^]^ (1.7.0). The peaks were added to gene expression matrix using FeatureMatrix and CreateChromatinAssay function in Signac package. All intersected peaks were filtered with a width threshold of less than 10,000 bp and greater than 20 bp. Seurat^[^ ^58^ ^]^ (4.3.0) was used to obtain nuclei with high qualities of both RNA‐seq and ATAC‐seq (nCount_ATAC < 100,000, nCount_RNA < 25,000, nCount_ATAC > 1,000, nCount_RNA > 1,000, nucleosome_signal < 2 and TSS.enrichment > 1).

### Processing and Normalization Steps for scRNA‐seq and scATAC‐seq Data were Performed as follows

NormalizeData, FindVariableFeatures, ScaleData RunPCA were used for scRNA‐seq, while in scATAC‐seq RunTFIDF, and FindTopFeatures (min.cutoff = “q0”) and RunSVD were applied. Reduction and clustering were performed by Seurat (4.3.0). Clusters for RNA‐seq and ATAC‐seq data were achieved using RunUMAP function (Seurat). Twenty‐one original clusters were obtained for RNA‐seq data with resolution set to “0.6”, min.dist to “0.3”, umap.method to “uwot”, metric to “cosine”, and dims ranging from 1 to 30. For ATAC‐seq data, 9 original clusters were obtained using resolution of “1”, min.dist of “0.1”, umap.method of “umap‐learn”, reduction of “lsi”, metric of “correlation”, and dims ranging from 2 to 30. A gene activity assay was computed by counting fragments that intersected with both the gene body and a 2‐kilobase upstream region for each gene within every individual cell. This analysis was performed using the GeneActivity function within Signac.

Marker genes for annotation of cell types and their references are listed in Table S1 (Supporting Information). ClosestFeature function was used in Signac package to find the nearest gene to DARs before performing cell annotation.

### Differential Gene Analysis

Differentially expressed genes (DEGs) for RNA‐seq analysis were calculated using the functions FindAllMarkers or FindMarkers (Seurat) with the Wilcox test, using an average log_2_ fold‐change threshold of 0.25 and an adjusted *p‐*value of less than 0.05. Differential accessible chromatin regions (DACRs) and differential gene activities were calculated using the same functions with the LR test, an average log_2_ fold‐change threshold of 0.125, an adjusted *p*‐value less than 0.05, and ATAC‐seq count as the latent variable.

### Gene Expression and Activity Correlation Analysis

Genes were overlapped with both gene expression and gene activity counts detected. Average levels of the overlapped gene expression and gene activity across different cell types were calculated by AverageExpression function in Seurat. The correlation was calculated by R function cor with method set to “spearman”.

### Cell Identity Differential Abundance Analysis

Differences in cell proportion were observed between the stress(*n* = 10,974) and control(*n* = 5,696) samples. The fold change in cell proportion of different cell types was defined by dividing the ratio of this cell type to the total number of cells under stress conditions by the ratio of this cell type to the total number of cells under control conditions. Statistical test of different cell type proportions was computed by R package edgeR^[^ ^59^ ^]^ (3.40.2). The glmQLFTest function was used in edgeR to perform a likelihood ratio test on a generalized linear model (GLM) to fit the data with coef set to 2. The topTags function was finally used in edgeR with “adjust.method” set to “fdr” to extract the adjusted p‐values which showed statistical differences in cell proportions.

### Development Trajectory Analysis

QC cells, epidermal initial cells, epidermis cells, trichoblast cells, and atrichoblast cells were denoted as epidermis lineage cells. Trajectories of the epidermis lineage (*n* = 5,917) and all cells (*n* = 16,670) were constructed using CytoTRACE (0.3.3).^[^ ^60^ ^]^ Expression changes along pseudotime in stress/control samples were fitted by geom_smooth function in R package ggplot2^[^ ^61^ ^]^ (3.4.1), the lines represent the mean expression levels while the bands around the fitted lines represent the 95% confidence level interval for predictions with smoothing method set to “gam”. Statistical tests between expression levels of stress and control nuclei were performed by two sided t‐test with t.test function in R. Stress/control‐dependent trajectories of trichoblast and suberin synthetic endodermis nuclei were constructed using Monocle^[^ ^62^ ^]^ (2.22.0). First, size factors and gene‐wise dispersions were estimated using the estimateSizeFactors and estimateDispersions functions, respectively. Next, a differential expression analysis was performed using the differentialGeneTest function. Gene expression was compared between different treatments, specified by the fullModelFormulaStr argument. A false discovery rate threshold (*q*‐value) of 0.003 was set for trichoblast nuclei to identify genes that showed statistically significant differential expression and 0.01 for suberin synthetic endodermis nuclei respectively. The nuclei was then ordered based on their expression levels of the significant genes using setOrderingFilter. This allowed to identify the developmental trajectory of the nuclei. Finally, the dimensionality of the data was reduced to 2 dimensions using the reduceDimension function with the DDRTree method. Branch‐dependent differentially expressed genes were calculated by BEAM function and were clustered into 3 groups.

### Calculation of Average Gene Expression and Chromatin Accessibility Levels in Cell Types

The average gene expression and gene activity levels in different cell types of different conditions were calculated by “AverageExpression” function with data from RNA assay and gene activity assays in Seurat. For each gene, the expression/activity levels were min‐max normalized so that the values were unified into the range of 0 to 1. The heatmaps for visualization were generated by “Heatmap” function in R package ComplexHeatmap (2.18.0).^[^ ^63^ ^]^

### Gene Ontology Enrichment Analysis

Gene Ontology enrichment analysis was achieved by enrichGO function from R package clusterProfiler^[^ ^64^ ^]^ (4.6.0) with ont set to “BP” and pAdjustMethod set to “BH”, only GO terms with adjust *p*‐values less than 0.05 were used for further analysis.

### Transcription Factor‐Target Network Construction

pySCENIC version 0.12.1^[^ ^65^ ^]^ was utilized to identify transcription factors (TFs) within trichoblast cells under two distinct experimental conditions. To construct the TF co‐expression network and access cisTarget databases specific to *Arabidopsis*, data were leveraged from scPlant.^[^ ^66^ ^]^ By cross‐referencing the TF lists obtained from scPlant with those from PlantTFDB,^[^ ^67^ ^]^ A consensus set of 674 *Arabidopsis* TFs was established, which served as the foundation for the subsequent analysis.

The initial co‐expression network between TFs and their potential target genes was generated from raw count matrices using the ‘pyscenic grn’ function. This preliminary network was refined in a subsequent step, where only the direct target genes were retained that possessed corresponding binding motifs for the regulating TF. This refinement process was executed using ‘pyscenic ctx.’ Each regulon, comprising a specific TF and its direct target genes, was identified and characterized. The activity of these regulons was quantified and binarized using the ‘pyscenic aucell’ function. Finally, the selected network was visualized using Cytoscape version 3.9.1.^[^ ^68^ ^]^ The target gene set scores were generated by AddModuleScore function in Seurat. The statistical differences of target gene set expression scores were performed by two‐sided wilcox tests.

### Quantitative Real‐Time PCR (RT‐qPCR)

*Arabidopsis* ecotype Col‐0 was cultured for 10 days in 1/2MS (control) and 1/2MS+250 mm sorbitol (stress) medium (21 °C, 16 h light/8 h dark cycle, 300 lux light intensity). Root tips(5mm) were collected for total RNA extraction. Total RNA was extracted by using an Rneasy Plant Mini kit (Qiagen). Then, these samples were performed by RT‐qPCR using SYBR Green Super mix (TOYOBO) on an Applied Biosystems 7,500 Fast Real‐Time PCR system. The primers used in these analyses as below:

*WRKY17*‐F: GGTTAGCTTCGTTCAGGCAA
*WRKY17*‐R: TTAGAGACACTTCCATCACC
*WRKY11*‐F: AAGAGATGTCTCGAGCATGA
*WRKY11*‐R: AATATTCGTCCGGTGGAATA
*Actin*‐F: GCACCCTGTTCTTCTTACCG
*Actin*‐R: AACCCTCGTAGATTGGCACA

### Gene‐Linked Candidate cis‐Regulatory Elements (gl‐cCREs) Identification and Hot Point gl‐cCREs Selection

*cis*‐co‐accessible networks with Cicero (version 1.3.9) was constructed from the scATAC‐seq peak counts. The Seurat object was transformed into the CellDataSet (CDS) format of Monocle3 (version 1.3.1) using the ‘as.cell_data_set’ function from the SeuratWrappers package.^[^ ^69^ ^]^ The Cell‐Cell Association Network (CCAN) was calculated using the ‘run_cicero’ and ‘generate_ccans’ function. The co‐accessibility of peaks under stress and control conditions was calculated separately and only those with “coaccess” score greater than 0.1 were kept. Next, the peak with promoter annotation in one co‐accessible peak pair was defined as the target peak, while the other peak became the cCRE. Then, co‐accessible peak pairs with significant positive correlation were kept between the accessibility of cCREs and the nearest gene expression of its target peak (correlation > 0.5, *p*‐value < 0.05). To discover stress‐related gl‐cCREs in each cell type, gl‐cCREs were compared from stress and control conditions. It was focused on gl‐cCREs that exclusively emerged under stress conditions, and visualized the outcome of this comparison through Venn diagrams. Stress‐related hgl‐cCREs were identified by selecting co‐access time outliers (more than three‐quarter quartile + 1.5* interquartile range IQR) from gl‐cCREs in different cell types. hgl‐cCREs peaks were demonstrated with CoveragePlot function in Signac.

## Conflict of Interest

The authors declare no conflict of interest.

## Author Contributions

Q.L., W.M., R.C., and S‐T.L. contributed equally to this work (co‐first authors). J.K., J.Z., Q.C., and H.‐X.S. are all co‐last authors. J.K., J.Z., Q.L., and H.‐X.S. conceived and designed the study. Q.L., W.M., and Q.W. performed the experiment. R.C., H.‐X.S., S‐T.L., Q.L., and J.K. and analyzed the data. Q.L., S‐T.L., H.‐X.S., and J.K. prepared the manuscript. J.Z., Q.C., and Y.H. revised the manuscript. All authors reviewed the manuscript.

## Supporting information

Supporting Information

Supplemental Table 1

Supplemental Table 2

Supplemental Table 3

Supplemental Table 4

Supplemental Table 5

Supplemental Table 6

Supplemental Table 7

Supplemental Table 8

Supplemental Table 9

## Data Availability

The raw data reported in this paper have been deposited in the NCBI GEO with the accession number GSE235510.
