# Supplementary material for: Multiome in the Same Cell Reveals the Impact of Osmotic Stress on Arabidopsis Root Tip Development at Single‐Cell Level
Source: Adv Sci (Weinh). 2024 Apr 18;11(24):2308384. doi: 10.1002/advs.202308384 (PMC11199978; doi:10.1002/advs.202308384)
Supplement: Supplementary file 1 — Supporting Information [file ADVS-11-2308384-s006.pdf]

ADVANCED SCIENCE
Open Access

Supporting Information

for *Adv. Sci.*, DOI 10.1002/advs.202308384

Multiome in the Same Cell Reveals the Impact of Osmotic Stress on *Arabidopsis* Root Tip Development at Single-Cell Level

Qing Liu, Wei Ma, Ruiying Chen, Shang-Tong Li, Qifan Wang, Cai Wei, Yiguo Hong, Hai-Xi Sun, Qi Cheng*, Jianjun Zhao* and Jingmin Kang**

Supporting Information

Multiome in the Same Cell Reveals the Impact of Osmotic Stress on *Arabidopsis* Root Tip Development at Single-Cell Level

Qing Liu[1], Wei Ma[1], Ruiying Chen[2,3,4], Shang-Tong Li[5], Qifan Wang[1], Cai Wei[2], Yiguo Hong[1,6], Hai-Xi Sun[2,3,4,], Qi Cheng[1,*], Jianjun Zhao[1,*], Jingmin Kang[2,3,*]*

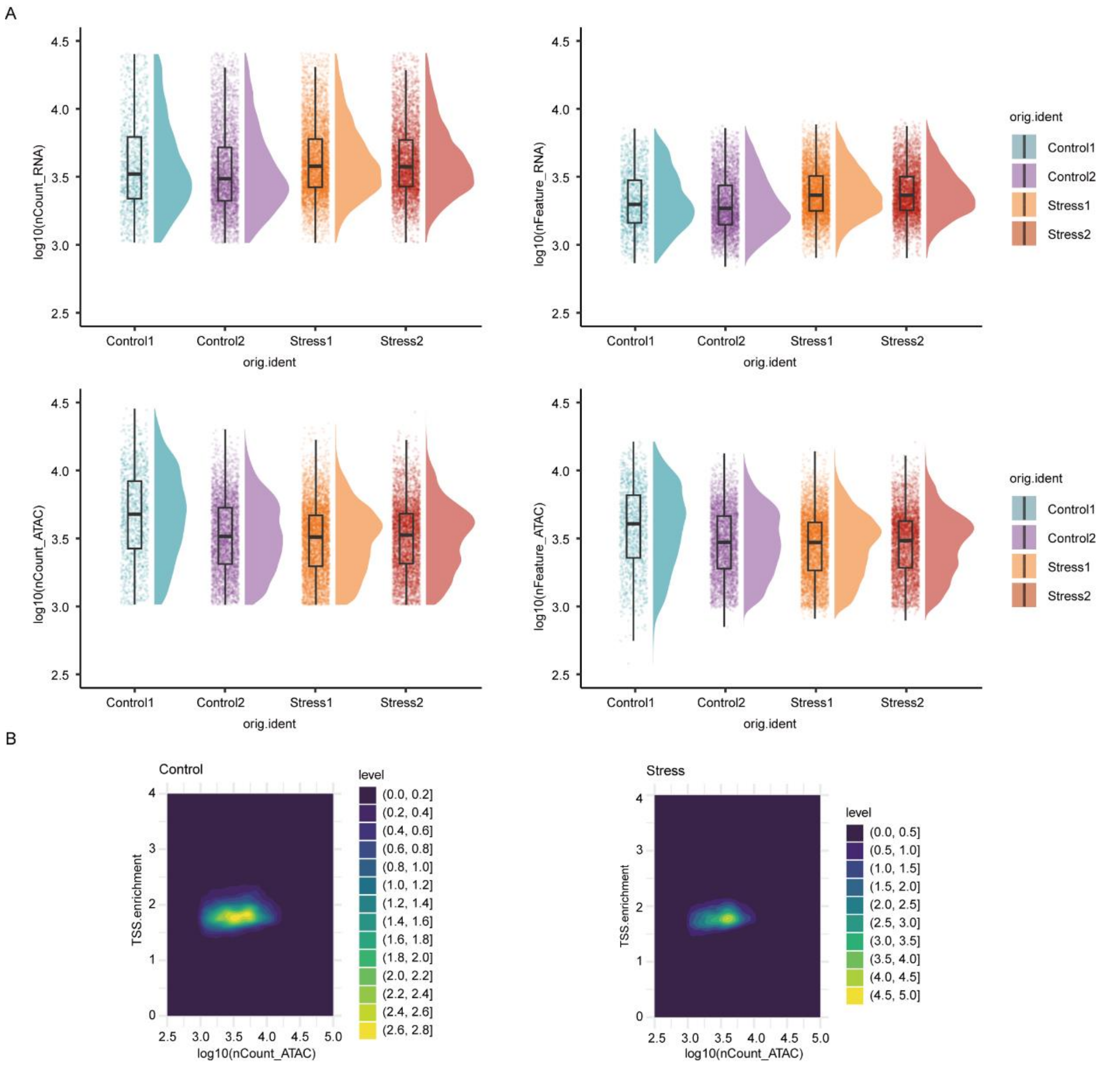

Figure S1 Estimation of the data quality. A) Scatter, box plot, and half-violin plots of $\log_{10}$(nCount_RNA), $\log_{10}$(nFeature_RNA), $\log_{10}$(nCount_ATAC), $\log_{10}$(nFeature_ATAC) in different samples. All four samples demonstrate a notable

level of quality that can be reasonably compared. B) Density contours of the cell distribution of the snATAC-seq quality control metrics (TSS enrichment and log_{10}(nCount_ATAC)) across control and stress samples. The density of the distribution was visualized with the use of color gradient.

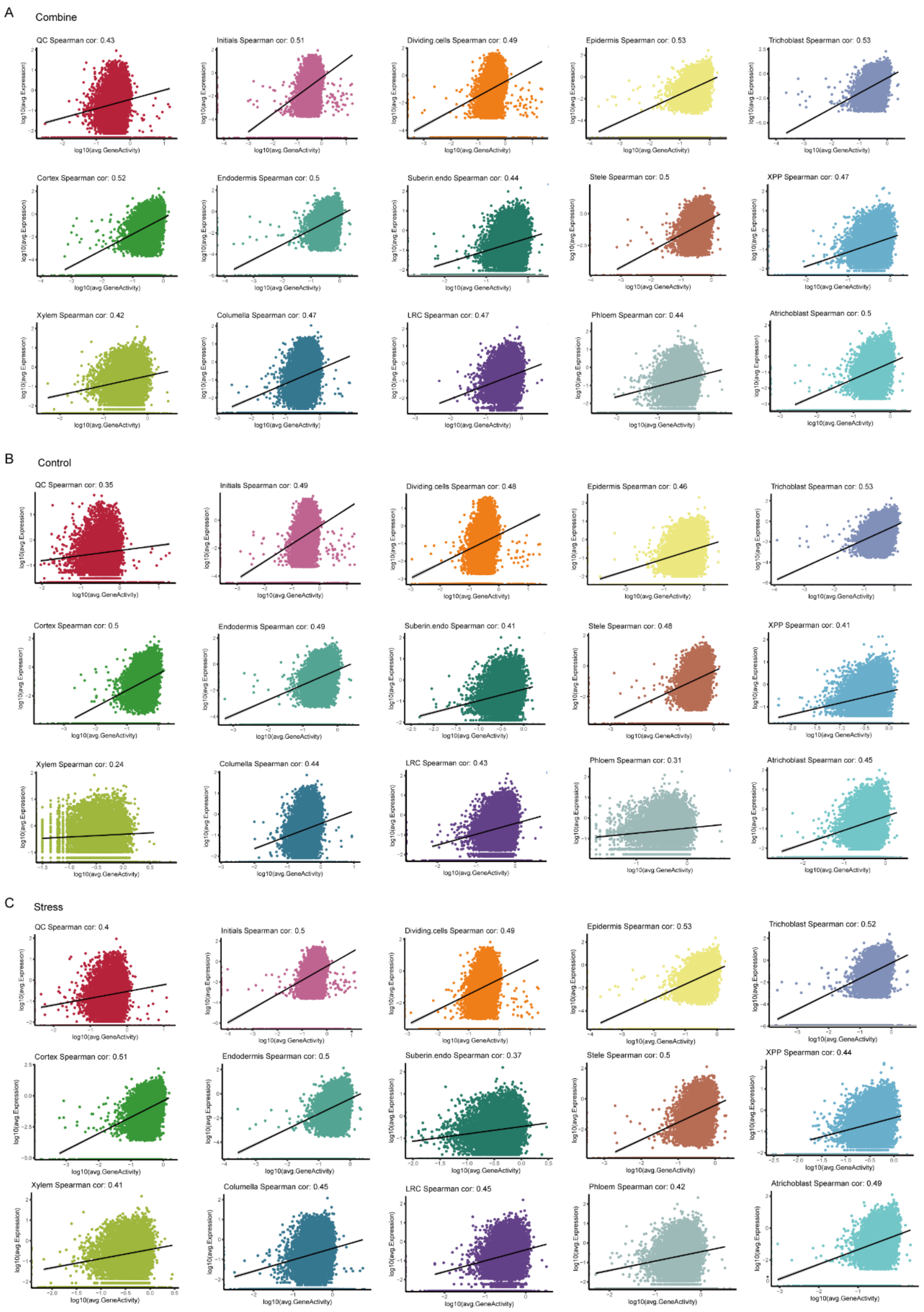

Figure. S2 Scatter plots showing the correlations between gene expression and gene activity for each cell type in combine(A), control(B) and stress(C) conditions, respectively.

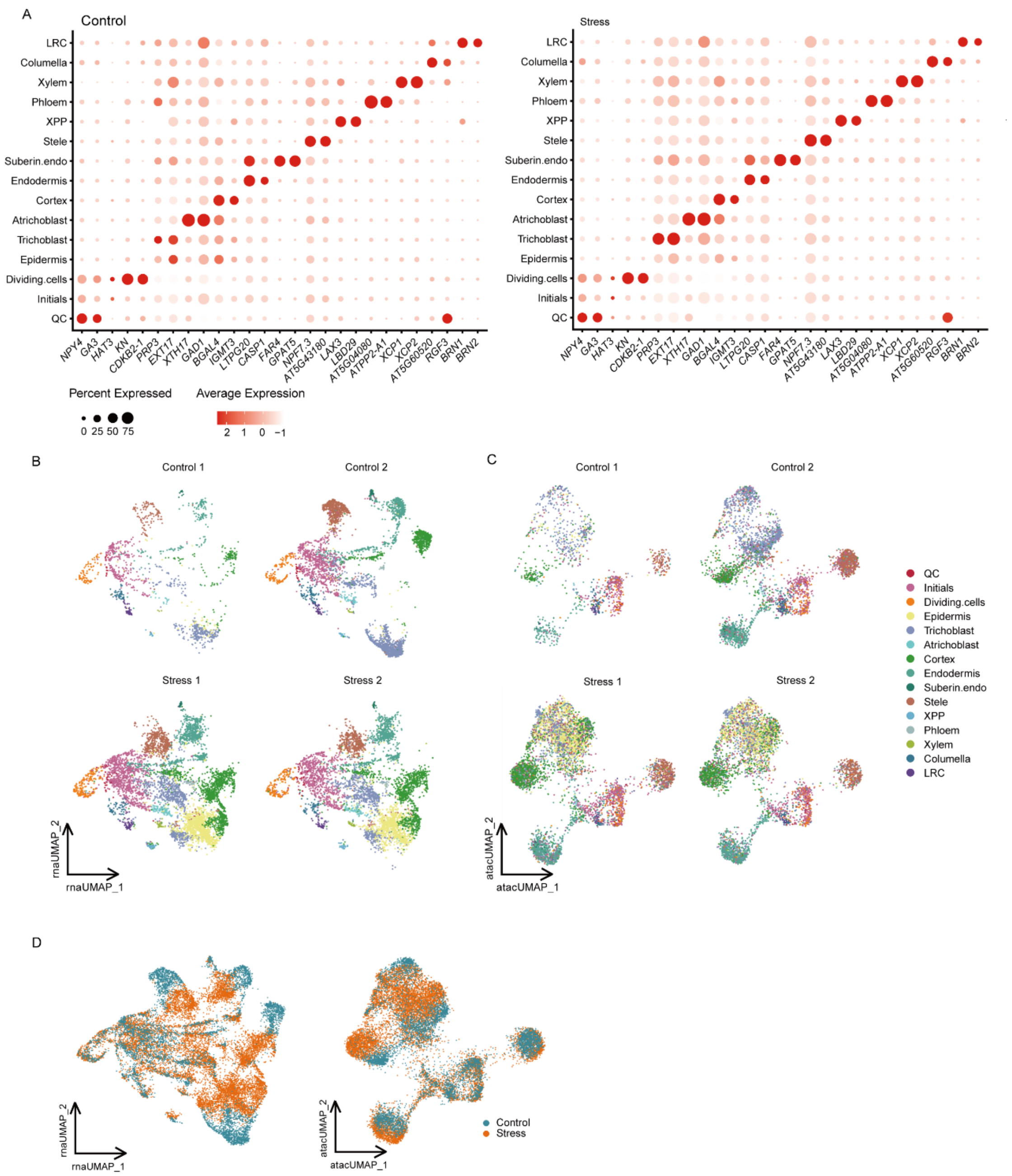

Figure. S3 A) Expression of known marker genes for each cell type in control(left) and stress(right) samples. Dot size indicates the percentage of cells expressing the gene (% expressed). B-C) UMAP visualization of two biological replicates in the control and stress group defined by RNA (B) and ATAC (C) annotation. D) RNA annotation (left) and ATAC annotation (right) with cells colored by control and stress conditions.

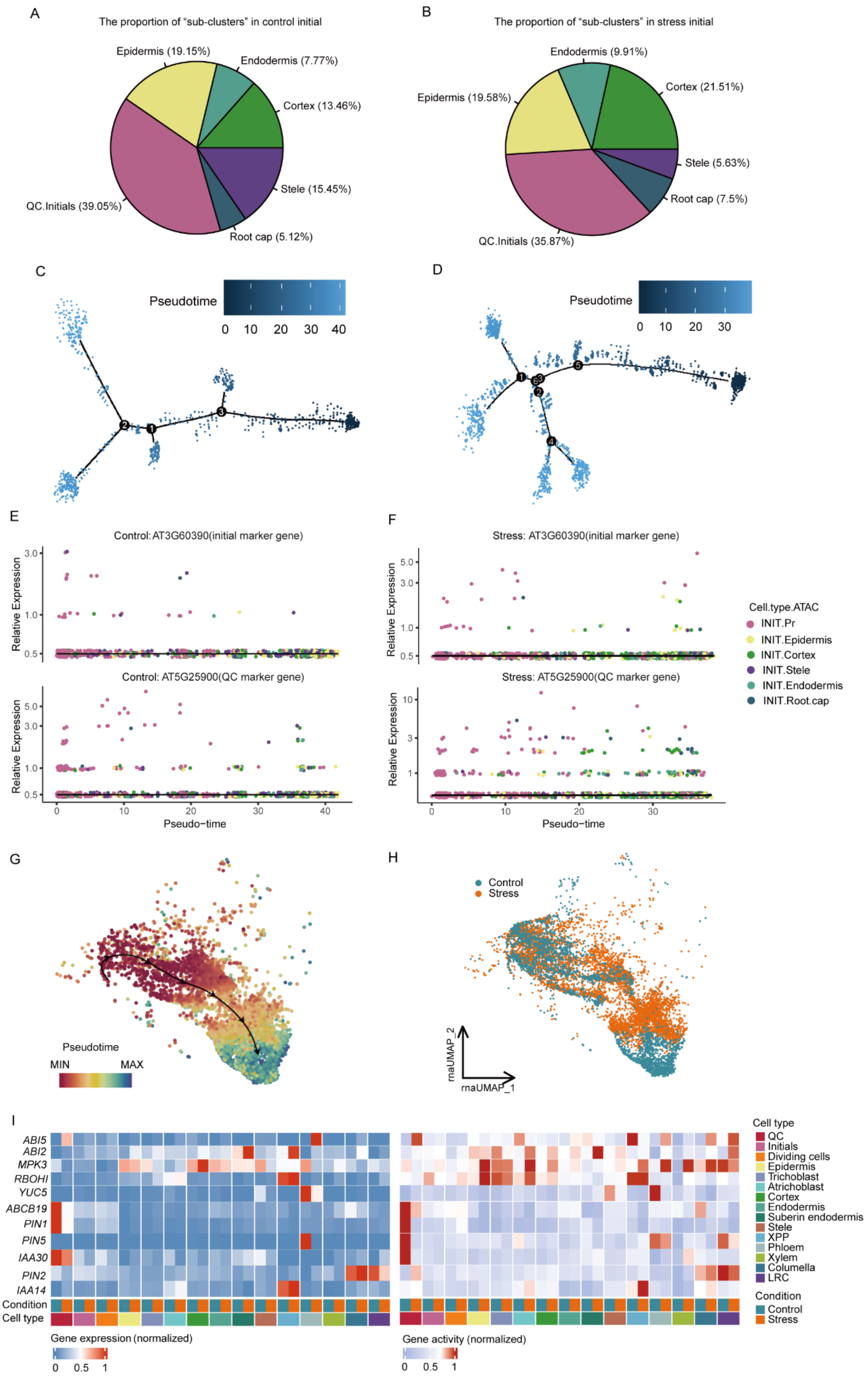

29 **Figure. S4** A-B) The proportion of "sub-clusters" in control (A) and stress (B) samples.

C-D) Developmental trajectories of control (C) and stress (D) initial cells (RNA annotation). Cells were colored by pseudo-time. E-F) Expression levels of QC and initial marker genes along pseudo-time trajectories. G-H) QC, initial, epidermis and trichoblast cells annotated with consensus pseudo-time levels (G) and culture conditions (H). I) Heatmap showing the average gene expression and gene activity levels in different cell types of different conditions.

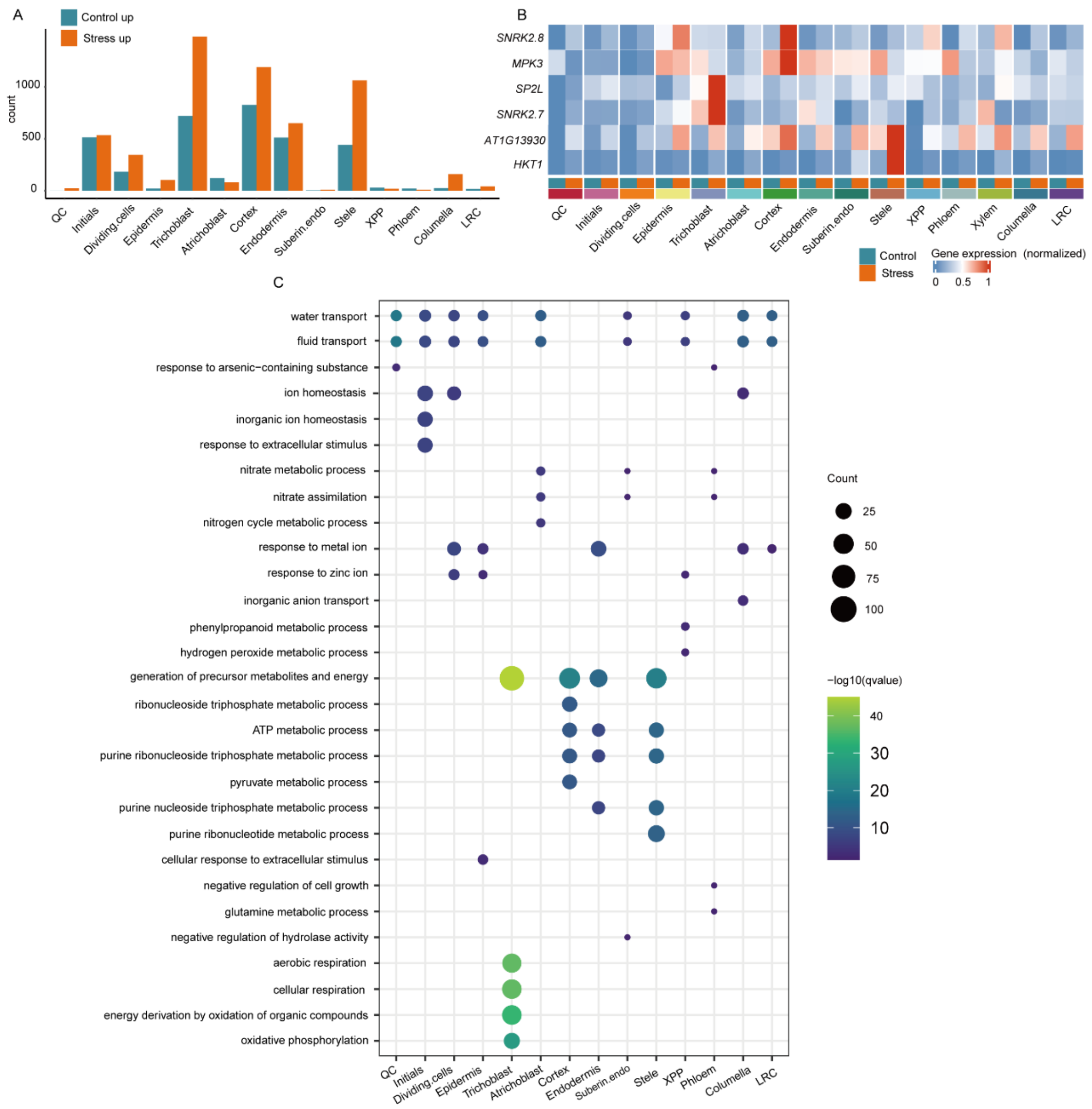

Figure. S5 A) Histogram showing the number of genes up-regulated in control and stress groups for each cell type, as calculated from the transcriptome data. B) Heatmap showing the master gene expression levels in different cell types of different conditions. C) Dot plots of GO enrichment analysis of different genes for each cluster.

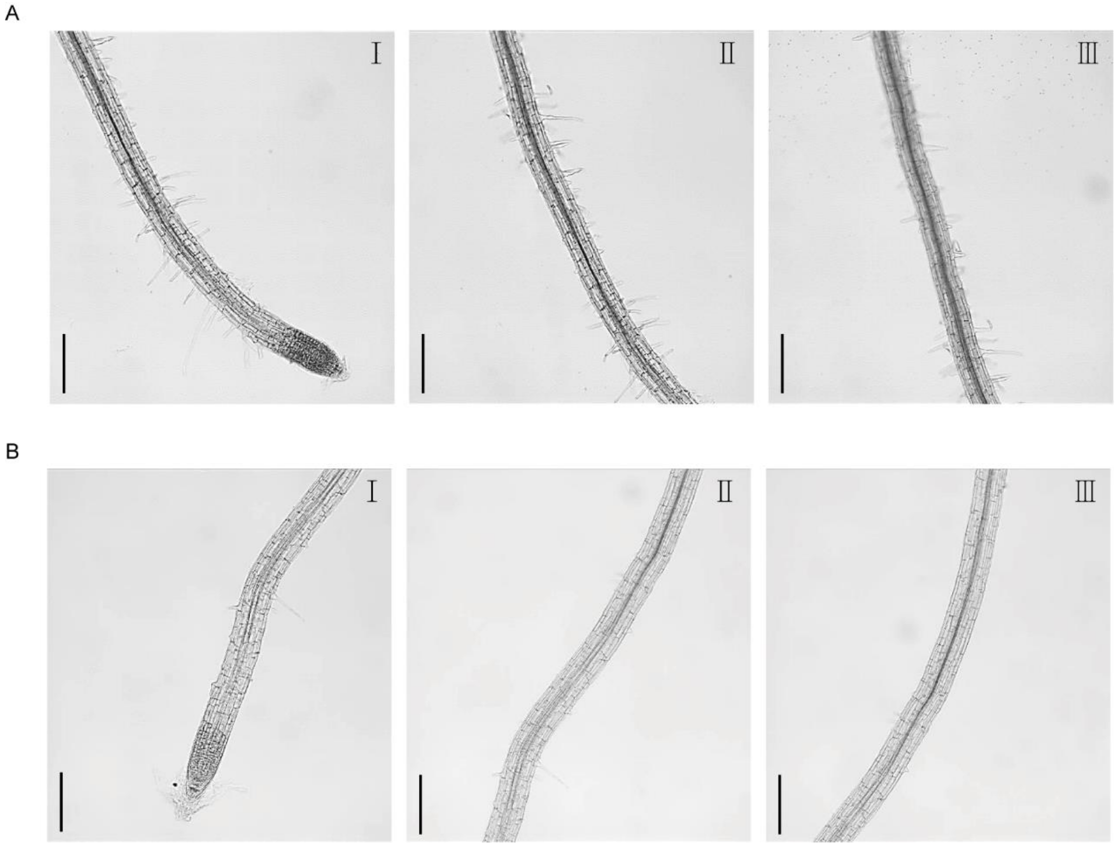

Figure. S6 Trichoblast phenotype of Col-0 root tips in different culture conditions. A-B) *Arabidopsis thaliana* ecotype Col-0 was grown in solid 1/2MS (control; A) and 1/2MS+250 mM sorbitol (stress; B) plates for 10 days (21℃ with 16h light/8h dark cycles, with light intensity of 300 lux).[24] Scale bars, 1cm. The Roman numerals I、II、III respectively represent successive spatial positions of the same root.

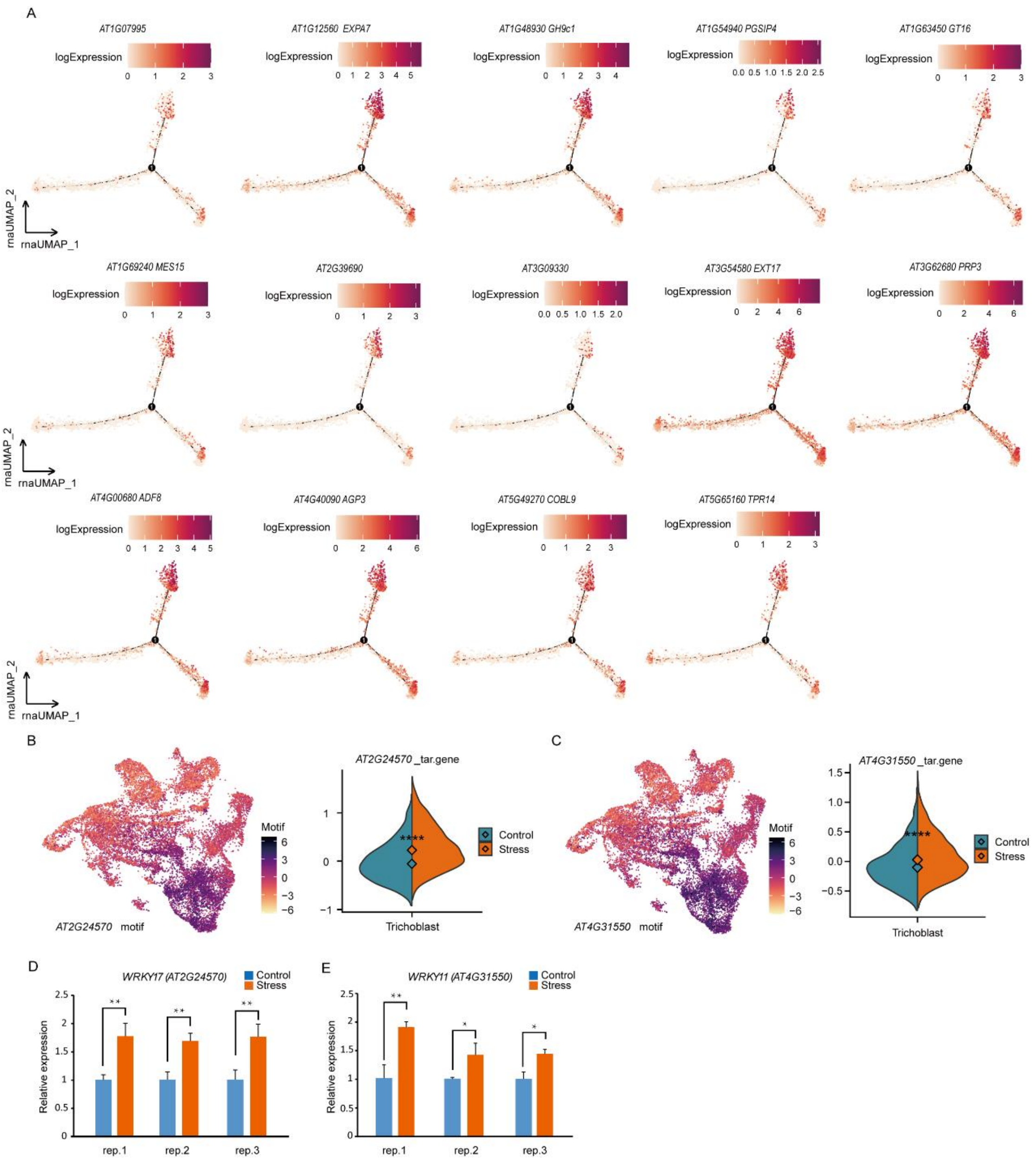

Figure. S7 A) Expression of trichoblast marker genes on the trichoblast pseudotime trajectory. The colors represent expression levels of these genes in individual cells. B-C) UMAP visualization of WRKY17(B) and WRKY11(C) motif accessibility(left) and violin plot of WRKY17(B) and WRKY11(C) target genes expression (right). D-E) The expression of *WRKY17*(D) and *WRKY11*(E) in wild-type root tips of control and osmotic stress samples. Error bars, means ± SD.

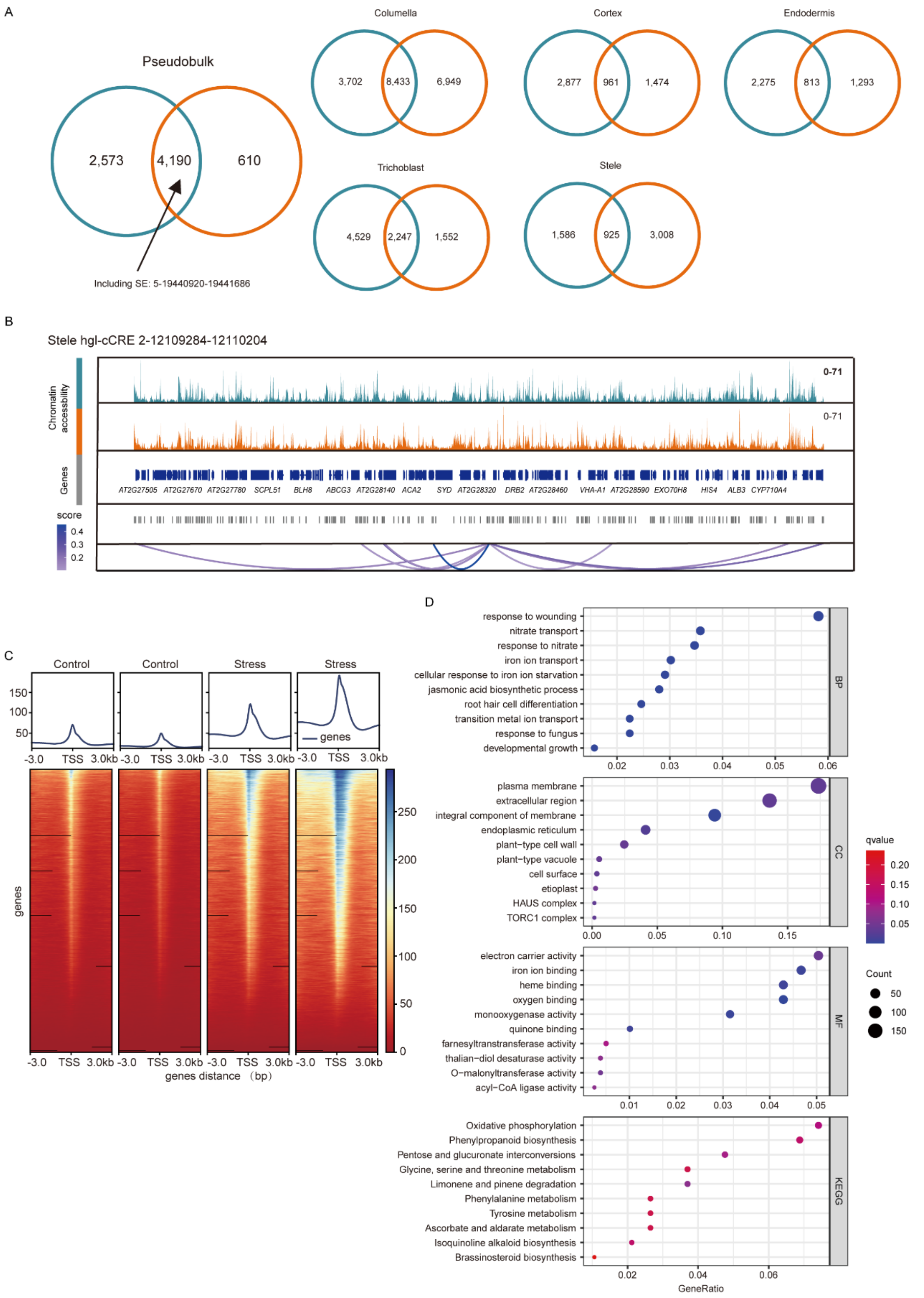

Figure. S8 A) Venn diagrams showing overlap of gl-cCREs under control and stress conditions. B) Accessibility level in different conditions and coaccessed peaks of stele hgl-cCRE 2-12109284-12110204. C) Heatmaps of individual samples were drawn using deeptools. Specifically, CUT&Tag signals 3Kb upstream and downstream of the

TSS were shown, where the signal intensities were ranked from high to low. D) BP, CC, MF and KEGG enrichment results of stress compared to control cells.
